# Unsupervised Chunking Based on Graph Propagation from Bilingual Corpus

**DOI:** 10.1155/2014/401943

**Published:** 2014-03-19

**Authors:** Ling Zhu, Derek F. Wong, Lidia S. Chao

**Affiliations:** Natural Language Processing & Portuguese-Chinese Machine Translation Laboratory, Department of Computer and Information Science, University of Macau, Macau

## Abstract

This paper presents a novel approach for unsupervised shallow parsing model trained on the unannotated Chinese text of parallel Chinese-English corpus. In this approach, no information of the Chinese side is applied. The exploitation of graph-based label propagation for bilingual knowledge transfer, along with an application of using the projected labels as features in unsupervised model, contributes to a better performance. The experimental comparisons with the state-of-the-art algorithms show that the proposed approach is able to achieve impressive higher accuracy in terms of *F*-score.

## 1. Introduction

Shallow parsing, is also called text chunking, plays a significant role in natural language processing (NLP) community. It can be regarded as a classification task, as a process of training a classifier to segment a sentence and labeling each partitioned phrase with an accurate chunk tag. The classifier takes the words, their POS tags, and surrounding context as features of an instance, whereas the chunk tag is the class label. The goal of shallow parsing is to divide a sentence into labeled, nonoverlapping, and nonrecursive chunks based on different methodologies.

Plenty of classification algorithms have been applied in the field of shallow parsing. The models are broadly categorized into three types: rule-based chunking model, machine learning-based model, and memory-based model. Particularly, in recent decades we have witnessed the remarkable advancement of the state of the art on chunking task by applying supervised learning approaches. Supervised chunking model, for example, MEMs (maximum entropy models), which is employed by [1] solves the ambiguous tagging problem by training on a corpus to compute the probability information of each word in the input context and get a good performance.

Although supervised learning algorithms have resulted in the state of the art and high accuracy systems on varieties of tasks in the NLP domain, the performance in source-poor language is still unreasonable. A fundamental obstacle of statistical shallow parsing for the quantities of world's language is the shortage of annotated training data. Furthermore, the work of well-understand hand annotation has proved to be expansive and time consuming. For example, over one million dollars have been invested in the development of Penn Treebank [2], and the lack of developed Treebank and tagged corpus in the majority of languages illustrate that it is difficult to raise the investment for annotation projects. However, unannotated parallel text data is broadly available because of the explosive growth of multilingual website, news streams, and human translations of books and documents. These suggest that unsupervised methods appear to be a useful solution for inducing chunking taggers, as they do not need any annotated text for training. Unfortunately, the existing unsupervised chunking systems do not have a reasonable performance to make its practical usability questionable at best.

To bridge the gap of the accuracy between source-rich languages and source-poor languages, we would like to leverage the existing resources of source-rich languages like English when doing the shallow parsing for the source-poor foreign languages such as Chinese. In this work, the system assumes that there is no labeled data available for training but that we have access to parallel English data. This part of work is closest to [3], but there are two main differences to improve the weakness of dealing with the unaligned words.

First, a novel graph-based framework is applied to project syntactic information across language boundaries for several reasons as follows.

- Graphs can indicate complicated relationships between classes and instances. For instance, an ambiguous instance interest could belong to the class of both NP and VP.
- Chunks from multiple sources, such as different tree banks, web sites,and texts can be represented in a single graph.
- The paths of information propagation of graphs make it possible to explicit the potential common information among different instances. That is to say, the algorithm relies on the fact that neighbor cases should belong to the same class, and the relationships between data points are captured in the form of a similarity graph with vertices corresponding to the cases and edge weights to their similarity.

In the end, a bilingual graph is constructed to represent the relationships between English and Chinese.

Second, rather than directly using these projected labels as features in supervised training phases, we prefer to employ them in unsupervised model. Additionally, to facilitate bilingual unsupervised chunking research and standardize best practice, a tag set consists of eight universal chunk categories is proposed in this work.

The paper is organized as follows.  Section 2 introduces the overall approach of our work.  Section 3 focuses on how to establish the graph and carry out the label propagation.  Section 4 describes the unsupervised chunk induction and feature selection in detail. In Section 5, the evaluation results are presented followed by a conclusion to end this paper.

## 2. Approach Overview

The central aim of our work is to build a chunk tagger for Chinese sentences, assuming that there is an English chunk tagger and some parallel corpuses between these two languages. Hence, we can conclude that the emphasis of our approach is how to build a bilingual graph from the sentence-aligned Chinese-English corpus. Two types of vertices will be employed in our graph: trigram types are used on the Chinese side corresponding to different vertices, while on the English side the vertices are individual word types. During the graph construction, no labeled data is needed but does require two similarity functions. A cooccurrence based similarity function is applied to compute edge weights between Chinese trigrams. This function is designed to indicate the syntactical similarity of the middle words of the neighbor trigrams. A second similarity function, which leverages standard unsupervised word alignment statistics, is employed to establish a soft correspond between Chinese and English.

**Figure 1:**
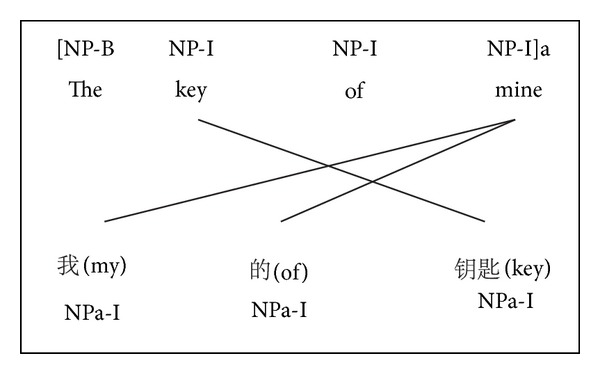
Direct label projection from English to Chinese with position information.

**Figure 2:**
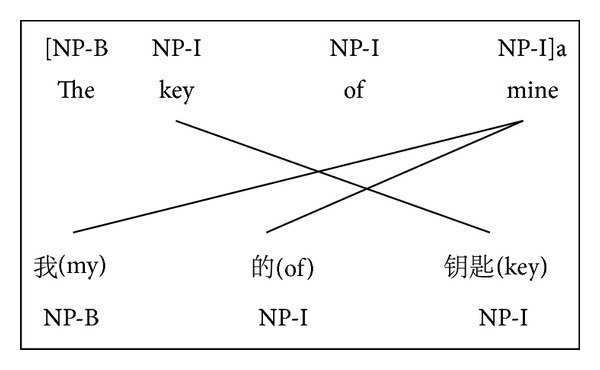
Adjust the Cchunk tag based on position information.

Based on the reality that we do not have labeled Chinese data, we aim to project the syntactic information from the English side to the Chinese side. Before initializing the graph, a supervised chunking model is used to tag the English side of the parallel text. The label distributions for the English vertices are generated by aggregating the chunk labels of the English tokens to types. Consider the crossover problem and different word orders between Chinese and English, the position information is also considered in our work. Based on this idea, the chunk tags are projected directly from English side to Chinese along with the position as shown in Figure 1. Based on the position information, we can get the exact boundary of each chunk at the Chinese side. Then an adjustment is made to assign the correct label as shown in Figure 2. After graph initialization, label propagation is applied to transfer the chunk tags to the peripheral Chinese vertices firstly (i.e., the ones which are adjacent to the English vertices), followed by further propagating among all the Chinese vertices. It is worth mentioning that the chunk distributions over the Chinese trigram types are regarded as features for learning a better unsupervised chunking tagger. The following sections will explain these steps in detail (see Algorithm 1).

## 3. Graph Construction

In graph learning approaches, one constructs a graph whose vertices are labeled and unlabeled examples and whose weighted edges encode the degree to which examples they link have the same label [4]. Notice that graph construction used for the problems of structured prediction such as shallow parsing is nontrivial for the following two reasons. First, it is necessary for resolving ambiguous problem by employing individual words as the vertices instead of the context. Besides, if we use the whole sentences as the vertices during graph construction, it is unclear how to calculate the sequence similarity. Altun et al. [5] proposed a graph-based semisupervised learning approach by using the similarity between labeled and unlabeled structured data in a discriminative framework. Recently, a graph over the cliques in an underlying structured model was defined by [6]. In their work, a new idea has been proposed that one can use a graph over trigram types, combing with the weights based on distribution similarity, to improve the supervised learning algorithms.

### 3.1. Graph Vertices

In this work, we extend the intuitions of [6] to set up the bilingual graph. Two different types of vertices are applied for each language because the information transformation in our graph is asymmetric (from English to Chinese). On the Chinese side, the vertices (denoted by *D* ^*c*^) correspond to trigram types, which are the same as in [6]. The English vertices (denoted by *D* ^*e*^), however, correspond to word types. The reason we do not need the trigram types in English side to disambiguate them is that each English word are going to be labeled. Additionally, in the label propagation or graph construction, we do not connect the vertices between any English words but only to the Chinese vertices.

Furthermore, there are two kinds of vertices consisting of ones extracted from the different sides of word aligned bitext (*D* ^*e*^, *D* ^*c*^) and an additional unlabeled Chinese monolingual corpus Γ^*c*^, which will be used later in the training phase. Such as noted, two different similarities will be employed to define the edge weights between vertices from these two languages and among the Chinese vertices.

### 3.2. Monolingual Similarity Function

In this section, a brief introduction is given as follows in terms of computing the monolingual similarity between the connecting pairs of Chinese trigram type. Specifically, the set *V* ^*c*^ consists of all the word *n*-grams that occur in the text. Given a symmetric similarity function between types defined below, we link types *m*_*i*_ and *m*_*j*_  (*m*_*i*_, *m*_*j*_ ∈ *V*_*c*_) with an edge weight *w*_*m*_*i*_*m*_*j*__ as follows:

(1) $$\begin{array}{c} w_{m_{i}m_{j}}=\{\begin{array}{cc} \text{sim}\left(m_{i},m_{j}\right) & \text{if}\;\;m_{i}\in \kappa \left(m_{j}\right)\;\;\text{or}\;\;m_{j}\in \kappa \left(m_{i}\right)\\ 0 & \text{otherwise}, \end{array} \end{array}$$

where *κ*(*m*_*j*_) is the set of *k*-nearest neighbors of *m*_*i*_ according to the given similarity. For all the parameters in this paper, we define *k* = 5.

To define the similarity function for each trigram type as *m*_*i*_ ∈ *V*_*c*_, and we rely on the cooccurrence statistics of ten features illustrated in Table 1.

Based on this monolingual similarity function, a nearest neighbor graph could be achieved. In this graph, the edge weight for the *n* most similar trigram types is set to the PMI values and is 0 for all other ones. Finally, we apply the function (*m*) to denote the neighborhood of vertex *m* and set the maximum number of 5 in our experiments.

### 3.3. Bilingual Similarity Function

We rely on high-confidence word alignments to define a similarity function between the English and Chinese vertices. Since the graph vertices are extracted from a parallel corpus, a standard word alignment technique GIZA++ [7] is applied to align the English sentences *D* ^*e*^ and their Chinese translations *D* ^*c*^. Based on the idea that the label propagation process in the graph will provide coverage and high recall, only a high confidence alignment *D* ^*e*↔*c*^ > 0.9 is considered.

Therefore, we can extract tuples of the form [*t*↔*u*] based on these high-confidence word alignments, where the Chinese trigram types that the middle word aligns to an English unigram type *u*. Relying on computing the proportion of these tuple counts, we set the edge weights between the two languages by our bilingual similarity function.

### 3.4. Graph Initialization

So far, we have introduced how to construct a graph. But the graph here is unlabeled completely. For the sake of label propagation, we have to use a supervised English chunking tagger to label the English side of the parallel corpus in the graph initialization phase. Afterwards we simply count the individual labels of each English token and then normalize the counts to get the tag distribution over the English unigram types. These distributions are in the use of initializing the English vertices' distribution in the graph. Considering that we extract the English vertices from a bitext, all vertices in English word types are assigned with an initial label distribution.

### 3.5. Graph Example

**Figure 3:**
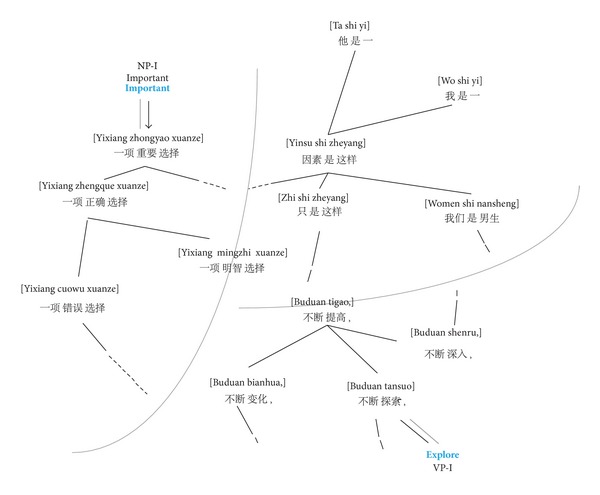
An example of similarity graph over trigram on labeled and unlabeled data.

Figure 3 shows a simple small excerpt from a Chinese-English graph. We see that only the Chinese trigrams [*一项* *重要* *选择*], [*因素* *是* *这样*] and [*不断* *深入*] are connected to the English translated words. All these English vertices are labeled by a supervised tagger. In this particular case, the neighborhoods can be more diverse and a soft label distribution is allowed over these vertices. As one can see, Figure 3 is composed of three subgraphs. In each subgraph, it is worth noting that the middle of Chinese trigram types has the same chunking types (with the labeled one). This exhibits the fact that the monolingual similarity function guarantees the connected vertices having the same syntactic category. The label propagation process then spreads the English words' tags to the corresponding Chinese trigram vertices. After that, labels are further propagated among the Chinese vertices. This kind of propagation is used to convey these tags inwards and results in tag distributions for the middle word for each Chinese trigram type. More details on how to project the chunks and propagate the labels will be described in the following section.

### 3.6. Label Propagation

Label propagation operates on the constructed graph. The primary objective is to propagate labels from a few labeled vertices to the entire graph by optimizing a loss function based on the constraints or properties derived from the graph, for example, smoothness [4, 8] or sparsity [9]. State-of-the-art label propagation algorithms include LP-ZGL [4], adsorption [10], MAD [8], and sparsity-inducing penalties [9].

Label propagation is applied in two phases to generate soft label distributions over all the vertices in the bilingual graph. In the first stage, the label propagation is used to project the English chunk labels to the Chinese side. In detail, we simply transfer the label distributions from the English word types to the connected Chinese vertices (i.e.,  *V*_*c*_^*l*^) at the periphery of the graph. Note that not all the Chinese vertices are fully connected with English words if we consider only the high-confidence alignments. In this stage, a tag distribution *d*_*i*_ over the labels *y* is generated, which represents the proportion of times the center word *c*_*i*_ ∈ *V*_*c*_ aligns to English words *e*_*y*_ tagged with label *y*:

(2) $$\begin{array}{c} d_{i}\left(y\right)=\frac{\sum_{e_{y^{}}}\#\left[c_{i}⟷e_{y}\right]}{\sum_{y^{\prime }}\sum_{e_{y^{\prime }}}\#\left[c_{i}⟷e_{y^{\prime }}\right]}. \end{array}$$

The second phase is the conventional label propagation to propagate the labels from the peripheral vertices *V*_*c*_^*l*^ to all Chinese vertices in the constructed graph. The optimization on the similarity graph is based on the objective function:

(3) P(q)=∑ci∈Vc∖Cclwij||qi−qj||2+λ||qi−U||2s.t.∑yqi(y)=1 ∀ciqi(y)≥0 ∀ci,yqi=di ∀ci∈Vcl,

where *q*_*i*_  (*i* = 1,…, |*V*_*c*_|) are the label distributions over all Chinese vertices and *λ* is the hyperparameter that will be discussed in Section 4. Consider that ||*q*_*i*_−*q*_*j*_||^2^ = ∑_*y*_(*q*_*i*_(*y*)−*q*_*j*_(*y*))^2^ is a squared loss, which is used to penalize the neighbor vertices to make sure that the connected neighbors have different label distributions. Furthermore, the additional second part of the regulation makes it possible that the label distribution over all possible labels *y* is towards the uniform distribution *U*. All these show the fact that this objective function is convex.

As we know, the first term in (3) is a smoothness regularizer which can be used to encourage the similar vertices, which have the large *w*_*ij*_ in between, to be much more similar. Moreover, the second part is applied to regularize and encourage all marginal types to be uniform. Additionally, this term also ensures that the unlabeled converged marginal vertices will be uniform over all tags if these types do not have a path to any labeled vertices. This part makes it possible that the middle word of this kind unlabeled trigram takes on any possible tag as well.

However, although a closed form solution can be derived through the objective function mentioned above, it would be impossible without the inversion of the matrix of order |*V*_*c*_|. To solve this problem, we rely on an iterative update based algorithm instead. The formula is as follows:

(4) $$\begin{array}{c} q_{i}^{\left(m\right)}\left(y\right)=\{\begin{array}{cc} c_{i}\left(y\right), & \text{if}\;\;c_{i}\in V_{c}^{l}\\ \frac{\gamma_{i}\left(y\right)}{\kappa_{i}}, & \text{otherwise}, \end{array} \end{array}$$

where  ∀*c*_*i*_ ∈ *V*_*c*_∖*V*_*c*_^*l*^, *γ*_*i*_(*y*), and *k*_*i*_ are defined as follows:

(5) $$\begin{array}{c} \gamma_{i}\left(y\right)=\sum_{c_{j}\in N(c_{i})}w_{ij}q_{i}^{\left(m-1\right)}\left(y\right)+\lambda U\left(y\right),\\ k_{i}=\lambda +\sum_{c_{j}\in N(c_{i})}w_{ij}. \end{array}$$

This procedure will be processed 10 iterations in our experiment.

## 4. Unsupervised Chunk Induction

In Section 3, the bilingual graph construction is described such that English chunk tags can be projected to Chinese side by simple label propagation. This relies on the high-confidence word alignments. Many Chinese vertices could not be fully projected from the English words. To complement, label propagation is used to further propagate among the Chinese trigram types. This section introduces the usage of unsupervised approach to build a practical system in the task of shallow parsing. The implementation of how to establish the chunk tag identification system will be discussed and presented in detail.

### 4.1. Data Representation

#### 4.1.1. Universal Chunk Tag

The universal tag was first proposed by [11] that consists of twelve universal part-of-speech categories for the sake of evaluating their cross-lingual POS projection system for six different languages. In this work, we follow the idea but focus on the universal chunk tags between English and Chinese for several reasons. First, it is useful for building and evaluating unsupervised and cross-lingual chunk taggers. Additionally, taggers constructed based on universal tag set can result in a more reasonable comparison across different supervised chunking approaches. Since two kinds of tagging standards are applied for the different languages, it is vacuous to state that “shallow parsing for language *A* is much harder than that for language *B*” when the tag sets are incomparable. Finally, it also permits our model to train the chunk taggers with a common tag set across multiple languages which does not need to maintain language specific unification rules due to the differences in Treebank annotation guidelines.

The following compares the definition of tags used in the English Wall Street Journal (WSJ) [2] and the Chinese Penn Treebank (CTB) 7.0 [12] that are used in our experiment.

*(i) The Phrasal Tag of Chinese.* In the present corpus CTB7, each chunk is labeled with one syntactic category. A tag set of 13 categories is used to represent shallow parsing structural information as follows.

 ADJP—adjective phrase: phrase headed by an adjective.
 ADVP—adverbial phrase: phrasal category headed by an adverb.
  CLP—classifiers phrase: for example, (QP (CD *一*) (CLP (M *系列*))) (a series).
  DP—determiner phrase: in CTB7 corpus, a DP is a modifier of the NP if it occurs within a NP. For example, (NP (DP (DT *任何*) (NP (NN *人*))) (any people).
 DNP—phrase formed by XP plus (DEG *的*) (‘s) that modifies a NP; the XP can be an ADJP, DP, QP, NP, PP, or LCP.
 DVP—phrase formed by XP plus “*地* (-ly)” that modifies a VP.
 FRAG—fragment used to mark fragmented elements that cannot be built into a full structure by using null categories.
 LCP—used to mark phrases formed by a localizer and its complement. For example, (LCP (NP (NR *皖南*) (NN *事变*)) (LC *中*)) (in the Southern Anhui Incident).
 LST—list marker: numbered list, letters, or numbers which identify items in a list and their surrounding punctuation is labeled as LST.
 NP—noun phrases: phrasal category that includes all constituents depending on a head noun.
 PP—preposition phrase: phrasal category headed by a preposition.
 QP—quantifier phrase: used with NP, for example, (QP (CD *五百*) (CLP (M *辆*))) (500).
 VP—verb phrase: phrasal category headed by a verb.

*(ii) The Phrasal Tag of English*. In the respect English WSJ corpus, there are just eight categories: NP (noun phrase), PP (prepositional phrase), VP (verb phrase), ADVP (adverb phrase), ADJP (adjective phrase), SBAR (subordinating conjunction), PRT (particle), and INTJ (interjection).

As we can see, the criterion of Chinese CTB7 has a lot of significant differences from English WSJ corpus. Unlike the English Treebank, the fragment of Chinese continents is normally smaller. A chunk which aligned to a base-NP phrase in the English side could be divided into a QP tag, a CLP tag, and an NP tag. For example, after chunking, “*五百* *辆* *车* (500 cars)” will be tagged as (NP (QP (CD *五百*) (CLP (M *辆*))) (NN *车*)). But at the English side, “500 cars” will be just denoted with an NP tag. This nonuniform standard will result in a mismatch projection during the alignment phase and the difficulty of evaluation in the cross-lingual setting. To fix these problems, mappings are applied in the universal chunking tag set. The categoryies CLP, DP, and QP are merged into an NP phrase. That is to say, the phrase such as (NP (QP (CD *五百*) (CLP (M *辆*))) (NN *车*)), which corresponds to the English NP chunk “500 cars,” will be assigned with an NP tag in the universal chunk tag. Additionally, the phrases DNP and DVP could be included in the ADJP and ADVP tags, respectively.

On the English side, the occurrence of INTJ is 0% according to statistics. This evidence shows that we can ignore the INTJ chunk tag. Additionally, the words belong to a PRT tag that is always regarded as a VP in Chinese. For example, (PRT (RP up)) (NP (DET the) (NNS stairs)) is aligned to “*上楼梯*” in Chinese where “*上*” is a VP.

#### 4.1.2. IBO2 Chunk Tag

There are many ways to encode the phrase structure of a chunk tag, such as IBO1, IBO2, IOE1, and IOE2 [13]. The one used in this study is the IBO2 encoding. This format ensures that all initial words of different base phrases will receive a B tag, which is able to identify the boundaries of each chunk. In addition, two boundary types are defined as follows:

- B-*X*: represents the beginning of a chunk* X*;
- I-*X*: indicates a noninitial word in a phrase* X*;
- O: any words that are out of the boundary of any chunks.

Hence, the input Chinese sentence can be represented using these notations as follows:

 
*去年* (NP-B) *实现* (VP-B) *进出口* (NP-B) *总值* (NP-I) *达* (VP-B) *一千零九十八点二亿* (NP-B) *美元* (NP-I) _°_ (O)

Based on the above discussion, a tag set that consists of six universal chunk categories is proposed as shown in Table 2. While there are a lot of controversies about universal tags and what the exact tag set should be used, these six chunk categories are sufficient to embrace the most frequent chunk tags that exist in English and Chinese. Furthermore, a mapping from fine-grained chunk tags for these two kind languages to the universal chunk set has been developed. Hence, a dataset consisting of common chunk tag for English and Chinese is achieved by combining with the original Treebank data and the universal chunk tag set and mapping between these two languages.

### 4.2. Chunk Induction

The label distribution of Chinese word types *x* can be computed by marginalizing the chunk tag distribution of trigram types *c*_*i*_ = *x*_−1_ *x*_0_ *x*_+1_ over the left and right context as follows:

(6) $$\begin{array}{c} p\left(y\;|\;x\right)=\frac{\sum_{x_{-1}x_{+1}}q_{i}\left(y\right)}{\sum_{x_{-1}x_{+1},y^{\prime }}q_{i}\left(y^{\prime }\right)}. \end{array}$$

Then a set of possible tags *t*_*x*_(*y*) is extracted through a way that eliminates labels whose probability is below a threshold value *τ*:

(7) $$\begin{array}{c} t_{x}\left(y\right)=\{\begin{array}{cc} 1 & \text{if}\;\;p\left(y\;|\;x\right)\geq \tau \\ 0 & \text{otherwise}. \end{array} \end{array}$$

The way how to choose *τ* will be described in Section 5. Additionally, the vector *t*_*x*_ will cover every word in the Chinese trigram types and will be employed as features in the unsupervised Chinese shallow parsing.

Similar to the work of [14, 15], our chunk induction model is constructed on the feature-based hidden Markov model (HMM). A chunking HMM generates a sequence of words in order. In each generation step, based on the current chunk tag *z*_*i*_ conditionally, an observed word *x*_*i*_ and a hidden successor chunk tag *z*_*i*+1_ are generated independently. There are two types of conditional distributions in the model, emission and transition probabilities, which are both multinomial probability distributions. Given a sentence *x* and a state sequence *z*, a first-order Markov model defines a joint distribution as

(8) Pθ=(X=x,Z=z)=Pθ(Z1=z1) ·∏i=1|x|Pθ(Zi+1=zi+1 ∣ Zi=zi) ·Pθ(Xi=xi ∣ Zi=zi),

where the second part represents the transition probability and the following part is emission probability, which is different from the conventional Markov model where the feature-based model replaces the emission part with a log-linear model, such that

(9) $$\begin{array}{c} P_{\theta }\left(X=x,Z=z\right)=\frac{\exp\theta^{Τ}f\left(x,z\right)}{\sum_{x^{\prime }\in \text{Val}(X)}\exp\theta^{Τ}f\left(x,z\right)}, \end{array}$$

which corresponds to the entire Chinese vocabulary.

The advantage of using this locally normalized log-linear model is the ability of looking at various aspects of the observation *x* incorporating features of the observations. Then the model is trained by applying the following objective function:

(10) $$\begin{array}{c} ℒ\left(\theta \right)=\overset{N}{\underset{i=1}{\sum }}\log\sum_{Z}P\left(\theta \right)\left(X=x^{\left(i\right)},Z=z^{\left(i\right)}\right)-C{||\theta ||}^{2}. \end{array}$$

It is worth noting that this function includes marginalizing out all the sentence *x*'s and all possible configurations *z*, which leads to a nonconvex objective. We optimize this objective function by using L-BFGS, a quasi-Newton method proposed by Liu and Nocedal [16]. The evidences of past experiments show that this direct gradient algorithm performed better than using a feature-enhanced modification of the EM (expectation-maximization).

Moreover, this state-of-the-art model also has an advantage that makes it easy to experiment with various ways of incorporating the constraint feature into the log-linear model. This feature function *f*_*t*_ consists of the relevant information extracted from the smooth graph and eliminates the hidden states which are inconsistent with the threshold vector *t*_*x*_.

### 4.3. Unsupervised Chunking Features

The feature selection process in the unsupervised chunking model does affect the identification result. Feature selection is the task to identify an optimal feature subset that best represents the underlying classification problem, through filtering the irrelevant and redundant ones. Unlike the CoNLL-2000, we shared supervised chunking task which is using WSJ (the Wall Street Journal) corpus as background knowledge to construct the chunking model. Our corpus is composed of only words which do not contain any chunk information that enables us to form an optimal feature subset for the underlying chunk pattern. We use the set of features as the following feature templates. These are all coarse features on emission contexts that are extracted from words with certain orthographic properties. Only the basic feature is used for transitions. For any emission context with word *x* and tag *z*, we construct the following feature templates as shown in Table 3.

Box 1 illustrates the example about the feature subset. The feature set of each word in a sentence is a vector of 6 dimensions, which are the values of the corresponding features and the label indicates which kind of chunk label should the word belong to.

## 5. Experiments and Results

Before presenting the results of our experiments, the evaluation metrics, the datasets, and the baselines used for comparisons are firstly described.

### 5.1. Evaluation Metrics

The metrics for evaluating NP chunking model constitute precision rate, recall rate, and their harmonic mean *F*_1_-score. The tagging accuracy is defined as

(11) Tagging   Accuracy =The  number  of  correct  tagged  wordsThe  number  of  words.

Precision measures the percentage of labeled NP chunks that are correct. The number of correct tagged words includes both the correct boundaries of NP chunk and the correct label. The precision is therefore defined as

(12) Precision =The  number  of  correct  proposed  tagged  wordsThe  number  of  correct  chunk  tags.

Recall measures the percentage of NP chunks presented in the input sentence that are correctly identified by the system. Recall is depicted as below

(13) $$\begin{array}{c} \text{Recall}=\frac{\text{The}\;\;\text{number}\;\;\text{of}\;\;\text{correct}\;\;\text{proposed}\;\;\text{tagged}\;\;\text{words}}{\text{The}\;\;\text{number}\;\;\text{of}\;\;\text{current}\;\;\text{chunk}\;\;\text{tags}}. \end{array}$$

The *F*-measure illustrates a way to combine previous two measures into one metric. The formula of *F*-sore is defined as

(14) $$\begin{array}{c} F_{\beta \text{-score}}=\frac{\left(\beta^{2}+1\right)\times \text{Recall}\times \text{Precision}}{\beta^{2}\times \text{Recall}+\text{Precision}}, \end{array}$$

where  *β* is a parameter that weights the importance of recall and precision; when *β* = 1, precision and recall are equally important.

### 5.2. Dataset

To facilitate bilingual graph construction and unsupervised chunking experiment, two kinds of data sets are utilized in this work: (1) monolingual Treebank for Chinese chunk induction and (2) large amounts of parallel corpus of English and Chinese for bilingual graph construction and label propagation. For the monolingual Treebank data we rely on Penn Chinese Treebank 7.0 (CTB7) [12]. It consists of over one million words of annotated and parsed text from Chinese newswire, magazines, various broadcast news, and broadcast conversation programs, web newsgroups, and weblogs. The parallel data came from the UM corpus [17], which contains 100,000 pair of English-Chinese sentences. The training and testing sets are defined for the experiment and their corresponding statistic information is shown in Table 4. In addition, Table 4 also shows the detailed information of data used for bilingual graph construction including the parallel corpus for the tag projection and the label propagation of Chinese trigram.

### 5.3. Chunk Tagset and HMM States

As described, the universal chunk tag set is employed in our experiment. This set *U* consists of the following six coarse-grained tags: NP (noun phrase), PP (prepositional phrase), VP (verb phrase), ADVP (adverb phrase), ADJP (adjective phrase), and SBAR (subordinating conjunction). Although there might be some controversies about the exact definition of such a tag set, these 6 categories cover the most frequent chunk tags that exist in one form or another in both of English and Chinese corpora.

For each kind of languages under consideration, a mapping from the fine-grained language specific chunk tags in the Treebank to the universal chunk tags is provided. Hence, the model of unsupervised chunking is trained on the datasets labeled with the universal chunk tags.

In this work, the number of latent HMM states for Chinese is fixed to be a constant, which is the number of the universal chunk tags.

### 5.4. Various Models

To comprehensively probe our approach with a thorough analysis, we evaluated two baselines in addition to our graph-based algorithm. We were intentionally lenient with these two baselines.

*(i) Feature-HMM.* The first baseline is the vanilla feature-HMM proposed by [9], which apply L-BFGS to estimate the model parameters and use a greedy many-to-1 mapping for evaluation.

*(ii) Projection.* The direct projection serves as a second baseline. It incorporates the bilingual information by projecting chunk tags from English side to the aligned Chinese texts. Several rules were added to fix the unaligned problem. We trained a supervised feature-HMM with the same number of sentences from the bitext as it is in the Treebank.

*(iii) Our Graph-Based Model.* The full model uses both stages of label propagation (3) before extracting the constrain features. As a result, the label distribution of all Chinese word types was added in the constrain features.

### 5.5. Experimental Setup

The experimental platform is implemented based on two toolkits: Junto [18] and Ark [14, 15]. Junto is a Java-based label propagation toolkit for the bilingual graph construction. Ark is a Java-based package for the feature-HMM training and testing.

In the feature-HMM training, we need to set a few hyperparameters to minimize the number of free parameters in the model. *C* = 1.0 was used as regularization constant in (10) and trained L-BFGS for 1,000 iterations. Several threshold values for *τ* to extract the vector *t*_*x*_ were tried and it was found that 0.2 works best. It indicates that *τ* = 0.2 could be used for Chinese trigram types.

### 5.6. Results

Table 5 shows the complete set of results. As expected, the projection baseline is better than the feature-HMM for being able to benefit from the bilingual information. It greatly improves upon the monolingual baseline by 12% on *F*_1_-score. Comparing among the unsupervised approaches, the feature-HMM achieves only 50% of *F*_1_-score on the universal chunk tags. Overall, the graph-based model outperforms these two baselines. That is to say, the improvement of feature-HMM with the graph-based setting is statistically significant with respect to other models. Graph-based modal performs 24% better than the state-of-the-art feature-HMM and 12% better than the direct projection model.

## 6. Conclusion

This paper presents an unsupervised graph-based Chinese chunking by using label propagation for projecting chunk information across languages. Our results suggest that it is possible for unlabeled text to learn accurate chunk tags from the bitext, the data which has parallel English text. In the feature, we propose an alternative graph-based unsupervised approach on chunking for languages that are lacking ready-to-use resource.

## Figures and Tables

**Box 1:**
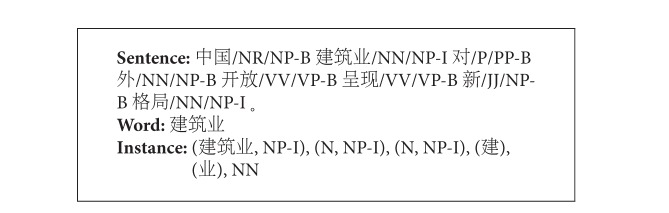
Example of feature template.

**Algorithm 1:**
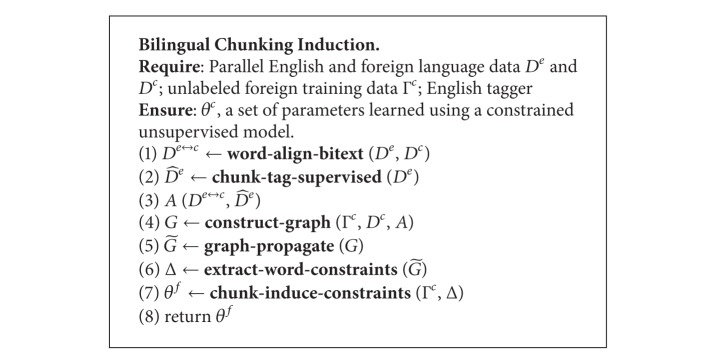
Graph-based unsupervised chunking approach.

**Table 1:** Various features for computing edge weights between Chinese trigram types.

Description	Feature
Trigram + context	*x*_1_ *x*_2_ *x*_3_ *x*_4_ *x*_5_
Trigram	*x*_2_ *x*_3_ *x*_4_
Left context	*x*_1_ *x*_2_
Right context	*x*_4_ *x*_5_
Center word	*x*_3_
Trigram − center word	*x*_2_ *x*_4_
Left word + right context	*x*_2_ *x*_4_ *x*_5_
Right word + left context	*x*_1_ *x*_2_ *x*_4_
Suffix	Has suffix (*x*_3_)
Prefix	Has prefix (*x*_3_)

**Table 2:** The description of universal chunk tags.

Tag	Description	Words	Example
NP	Noun phrase	DET + ADV + ADJ + NOUN	The strange birds
PP	Preposition phrase	TO + IN	In between
VP	Verb phrase	ADV + VB	Was looking
ADVP	Adverb phrase	ADV	Also
ADJP	Adjective phrase	CONJ + ADV + ADJ	Warm and cozy
SBAR	Subordinating conjunction	IN	Whether or not

**Table 3:** Feature template used in unsupervised chunking.

Basic:	(*x*=, *z* = )

Contains digit:	check if* x* contains digit and conjoin with *z* (contains digit(*x*)=, *z* = )

Contains hypen:	contains hypen(*x*)=, *z* =

Suffix:	indicator features for character suffixes of up to length 1 present in *x*

Prefix:	indicator features for character prefixes of up to length 1 present in *x*

Pos tag:	indicator feature for word POS assigned to *x*

**Table:** (a)

Number of sentence pairs	Number of seeds	Number of words
10,000	27,940	31,678

**Table:** (b)

Number of sentences	Number of vertices
17,617	185,441

**Table:** (c)

Dataset	Source	Number of sentences
Training dataset	Xinhua 1–321	7,617
Testing dataset	Xinhua 363–403	912

**Table 5:** Chunk tagging evaluation results for various baselines and proposed graph-based model.

Tag	Feature-HMM			Projection			Graph-based		
	Pre	Rec	F1	Pre	Rec	F1	Pre	Rec	F1
NP	0.60	0.67	0.63	0.67	0.72	0.68	0.81	0.82	0.81
VP	0.56	0.51	0.53	0.61	0.57	0.59	0.78	0.74	0.76
PP	0.36	0.28	0.32	0.44	0.31	0.36	0.60	0.51	0.55
ADVP	0.40	0.46	0.43	0.45	0.52	0.48	0.64	0.68	0.66
ADJP	0.47	0.53	0.50	0.48	0.58	0.51	0.67	0.71	0.69
SBAR	0.00	0.00	0.00	0.50	1.0	0.66	0.50	1.0	0.66
All	0.49	0.51	0.50	0.57	0.62	0.59	**0.73**	**0.75**	**0.74**
